# Amphioxus encodes the largest known family of green fluorescent proteins, which have diversified into distinct functional classes

**DOI:** 10.1186/1471-2148-9-77

**Published:** 2009-04-21

**Authors:** Erin K Bomati, Gerard Manning, Dimitri D Deheyn

**Affiliations:** 1 Scripps Institution of Oceanography, University of California San Diego, La Jolla, CA 92093, USA; 2 Razavi Newman Center for Bioinformatics, Salk Institute for Biological Studies, La Jolla, CA 92037, USA

## Abstract

**Background:**

Green fluorescent protein (GFP) has been found in a wide range of Cnidaria, a basal group of metazoans in which it is associated with pigmentation, fluorescence, and light absorbance. A GFP has been recently discovered in the pigmentless chordate *Branchiostoma floridae *(amphioxus) that shows intense fluorescence mainly in the head region.

**Results:**

The amphioxus genome encodes 16 closely-related GFP-like proteins, all of which appear to be under purifying selection. We divide them into 6 clades based on protein sequence identity and show that representatives of each clade have significant differences in fluorescence intensity, extinction coefficients, and absorption profiles. Furthermore, GFPs from two clades exhibit antioxidant capacity. We therefore propose that amphioxus GFPs have diversified their functions into fluorescence, redox, and perhaps just light absorption in relation to pigmentation and/or photoprotection.

**Conclusion:**

The rapid radiation of amphioxus GFP into clades with distinct functions and spectral properties reveals functional plasticity of the GFP core. The high sequence similarities between different clades provide a model system to map sequence variation to functional changes, to better understand and engineer GFP.

## Background

The discovery of green fluorescent protein (GFP) in the bioluminescent jellyfish *Aequorea victoria *[1] sparked the interest of marine ecologists, cell biologists, and spectroscopists alike. The subsequent cloning [2] and characterization [3,4] of GFP revealed that its energy-absorbing core, the chromophore, is self-generated via cyclization of a peptide triplet buried in the interior of a protective β-can protein fold [5,6]. Once oxidized using molecular oxygen, the chromophore shows high stability and absorbance of high-energy light (blue) that is efficiently re-emitted as fluorescence of lower-energy (green) light over a wide range of conditions. The ease of expression of GFP in a variety of hosts has enabled a myriad of fluorescence imaging applications, from quantifying transgene expression to probing enzyme activity and protein-protein interactions [7-10]. Its tremendous utility was recognized by the award of the 2008 Nobel prize in Chemistry.

Since the discovery of GFP in *Aequorea victoria*, researchers have identified GFP-like proteins from other cnidarians with distinctive biochemical and spectroscopic characteristics, extending their utility as fluorescence probes [11,12]. While the industrial race has been running strong to find new and innovative applications for GFP as well as to utilize protein engineering to push the boundaries of traditional GFP properties, evolutionary ecologists and marine biologists have continued to strive to understand the function(s) of GFP in marine organisms.

GFP-like proteins in non-bioluminescent Cnidaria have varying fluorescence peaks, from cyan to red [4], in some cases within the same individual. This observation prompts the question of how many GFP-like genes can be found co-occurring in one organism. While the three GFP isoforms in *Aequorea victoria *differ by only 4 amino acids and are thought to be population variants of a single gene [2], other organisms have multiple GFPs. In corals, two distinct GFP-like proteins were discovered from *Zoanthus *(zFP506 and zFP538) and two others from *Discosoma *(dsFP483 and drFP583). Despite only a few amino acid replacements between each pair, their emission spectra differ dramatically [13]. More recently four spectrally distinct GFP-like genes were reported within the great star coral, *Montastraea cavernosa *[14]. In reef building corals, it is believed that GFP-like proteins are the main determinants of pigment color [15,16]. The presence of multiple GFPs is addressed by the polyphenism model, which proposes that differential expression of multiple GFP genes offers a palette of pigment colors to adapt to physiological, ecological or developmental changes [8]. These colors can be derived from non-fluorescent GFPs which are deemed chromoproteins due to their internal chromophore that absorbs but generally does not re-emit light [15,17,18]. In addition to pigmentation, GFP has several other proposed light-driven functions including photoprotection [19], photoreception and enhancement of photosynthesis [20,21], as well as non-light driven functions such as radical scavenging [22]. Therefore, in some cnidarians selective pressure on pigment color may be the driving force for GFP evolution, while in other cnidarians selective pressure may have shifted to alternative functions of GFP. GFP has recently been found outside the Cnidaria, in protostome crustacean copepods [23] and the deuterostome chordate amphioxus [24,25]. The occurrence of GFP in these evolutionary distant non-bioluminescent organisms with distinct ecology from cnidarians is intriguing, and characterizing GFP in these organisms may provide insight into additional functions acquired by GFP through evolution.

Here, we identify and initially characterize a family of 16 GFP-like proteins in amphioxus (*Branchiostoma floridae*), the largest set of GFPs known in a single organism. This extensive family comprises proteins of drastically differing fluorescence intensities and absorbance spectra. We propose that some members have light-related functions with a true fluorescence outcome or with only efficient light absorption (e.g., for photoprotection, photoreception) while others have alternative biochemical functions through antioxidant mechanisms (e.g., for cellular defense).

## Results

### A family of 16 GFP genes in amphioxus

We identified 16 unique GFP-like genes within the amphioxus genome. Initial Blast and HMM searches of the predicted proteome [26] gave 26 predicted GFP-like sequences or fragments. Two predictions covered parts of the same gene, another prediction covered two neighboring genes, and another 10 appear to be allelic variants, and were denoted with an "a" suffix (Table 1). HMM searches of the genome assembly revealed a number of duplicate fragments within introns of the identified GFPs, but did not identify any new genes.

**Table 1:** Amphioxus GFP genes.

**Gene**	**JGI Gene Model**	**Baumann et al. 2008**	**Scaffold**	**Position**	**Correction of Gene Prediction**	**Gene Product**	**EST Count**
**Clade a**							

GFPa1	fgenesh2_pg.scaffold_1000062	Lan1	1	1116500–1118933	None	EST/PCR	204
GFPa2	fgenesh2_pg.scaffold_264000004	Lan3	264	58192–60413	Trimmed N-term	EST	27
GFPa3	fgenesh2_pg.scaffold_549000016	Lan9	549	352398–358639	None	No	
GFPa4	fgenesh2_pg.scaffold_549000017		549	364853–368343	Trimmed C-term	No	
GFPa5	fgenesh2_pg.scaffold_1000068	Lan12	1	1208185–1211151	Trimmed C-term	No	

**Clade b**							

GFPb1	fgenesh2_pg.scaffold_408000038	Lan13	408	704470–709647	Trimmed N-term	No	
GFPb1a	fgenesh2_pg.scaffold_58000035		58	532019–534945	Trimmed N-term (7AA + ATG)	No	
GFPb2	fgenesh2_pg. scaffold_408000037		408	691033–694888	Extended N, C term	No	
GFPb2a	fgenesh2_pg. scaffold_58000034	Lan19	58	518572–521504	Trimmed start (48AA)	No	
GFPb3	estExt_fgenesh2_pg.C_4080036	Lan5-Nterm	408	656826–660682	Extended N-term, trimmed C-term.	EST	1
GFPb3a	fgenesh2_pg.scaffold_58000032		58	485880–488855	Trimmmed N-term, added ATG	No	
GFPb4	estExt_fgenesh2_pg.C_4080036	Lan5-Cterm	408	674499–680280	Trimmed N-term.	EST	1
GFPb4a	fgenesh2_pg.scaffold_58000033		58	507562–508283	Two-exon fragment. Trimmed and extended both ends	No	

**Clade c**							

GFPc1	fgenesh2_pg.scaffold_722000001	Lan11	722	186–3347	Trimmed N-term, added ATG	No	
GFPc1a	fgenesh2_pg.scaffold_58000036		58	555111–557942	None	PCR	

**Clade d**							

GFPd1	fgenesh2_pg.scaffold_149000048		150	209871–211956	Trimmed N-term	EST/PCR	13
GFPd1a	fgenesh2_pg.scaffold_150000025		149	908671–910787	None	No	
GFPd2	fgenesh2_pg.scaffold_771000005		771	43072–45512	None	EST/PCR	5
GFPd2a	estExt_fgenesh2_pg.C_2370020	Lan7	237	340592–343047	Trimmed N-term	No	

**Clade e**							

GFPe1	fgenesh2_pg.scaffold_1000063		1	1141692–1143777	Filled internal deletion	EST/PCR	194
GFPe2	fgenesh2_pg.scaffold_1000066	Lan4	1	1168253–1170596	None	EST	26
GFPe2a	fgenesh2_pg.scaffold_13000140		13	3112741–3113363	Extended N-term	No	
GFPe3	fgenesh2_pg.scaffold_1000064	Lan2	1	1150911–1152721	None	EST	125
GFPe3a	fgenesh2_pg.scaffold_264000003		264	31164–32864	Extended C-term	No	

**Clade f**							

GFPf1	fgenesh2_pg.scaffold_1000065	Lan6	1	1159351–1162889	None	EST	3
GFPf1a	fgenesh2_pg.scaffold_264000002		264	14129–18055	Trimmed internal insert	EST (truncated)	

List of amphioxus GFP gene sequences with genomic mapping, errors in original gene predictions, evidence of gene product, and EST count. Secondary alleles have an "a" suffix. EST count refers to the number of GFP sequences in library of 334,502 *B. floridae *ESTs.

We used manual curation to extend and correct errors in several gene predictions (Table 1). Curation was aided by the absolutely conserved intron-exon structure and the presence of just 6 internal indels within the aligned proteins, verified by EST sequences from several genes. The first exon encodes only the starting methionine, and was predicted based on EST alignments and on conserved splice sites upstream of the second exon. The second alleles of three genes (GFP18a, GFP3a and GFP5a) are close to gaps in the assembly and remain as fragments.

BLAT mapping to the genomic assembly shows evidence of extensive tandem duplication: the three members of Clade E are within 29 kb of each other, the four Clade B genes are within 55 kb in each haplotype, and GFPa3 and GFPa4 are 15 kb apart. Many other genes map to short scaffolds. Our multiple cross-analyses provide an exhaustive list of the GFPs found in the amphioxus genome, thus completing an earlier report of 12 GFP-like proteins [24]. Those sequences are cross-referenced in Table 1.

**Figure 1:**
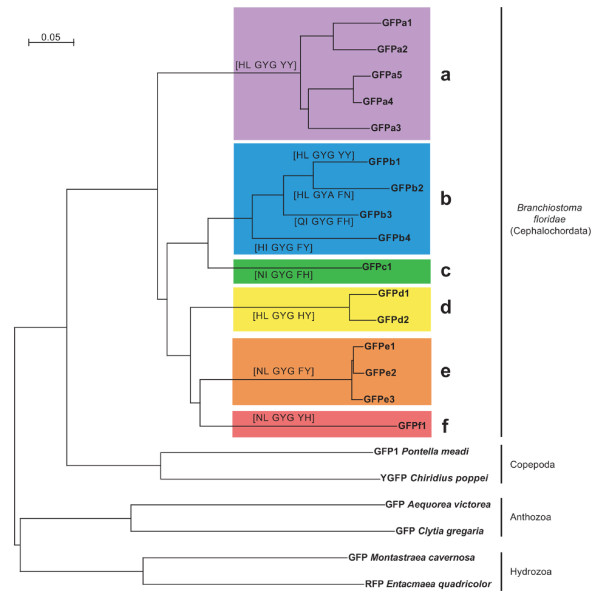
**Phylogenetic tree of amphioxus green fluorescent proteins**. GFP-like proteins are organized in 6 clades designated a – f. The chromophore region, encompassing the chromophore triplet and the two residues before and after the triplet, is shown in brackets. Representative GFPs from copepods (protostomes) and the anothozoan and hydrozoan cnidarians are also shown. Interspecies bootstraps are all 100%.

All genes are highly similar to each other, with >47% AA sequence identity, compared to <33% for the nearest homologs in other species. Sequence analysis places them in 6 clades, a to f (Fig. 1; clade names incorporated into gene names) of highly-related sequences. Proteins within the same clade have an average protein sequence identity of 91%, while average inter-clade identities ranged from 49% (a to e) to 65% (b to c). The synonymous substitution rate (Ks) between clade members is always <0.32, while all inter-clade comparisons had Ks >0.47 (Additional file 1), suggesting that clade members result from recent duplications. Apart from Clade b, each clade can be distinguished by the sequence of the chromophore triplet and surrounding pairs of residues (brackets in Fig. 1), which are known to influence the spectroscopic properties in other GFPs [27,28].

### Multiple GFPs from different clades are expressed in amphioxus

**Figure 2:**
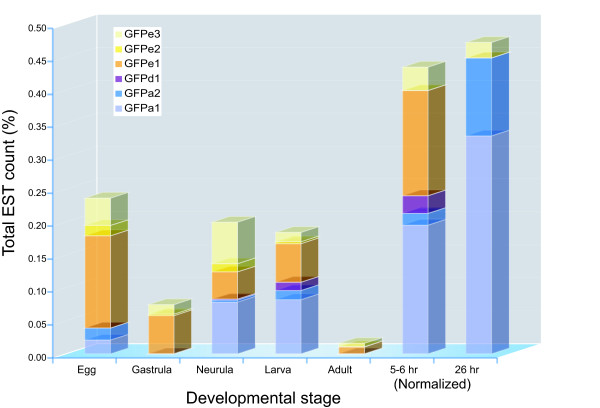
**Expression of GFPs in developmentally staged EST libraries**. The occurance of ESTs from selected GFPs is shown as a percentage of total ESTs per stage, extracted from the dbEST database (Table S1). GFPs with <10 ESTs were omitted. The 5–6 (gastrula) and 24 hr (neurula) libraries were normalized using oligonucleotide fingerprinting, and do not accurately represent total EST concentrations.

Members of all clades were found to be expressed, based on EST sequences or RT-PCR (Table 1). ESTs were found for 10 genes, with most ESTs (523/599) from just three genes: GFPa1, GFPe1 and GFPe3 (Table S1). GFP expression is highly dynamic throughout the developmentally staged EST libraries, and each expressed GFP shows a distinct time-course of expression (Fig. 2). The 5–6 and 26 hour libraries correspond to gastrula and neurula stages, respectively, but were normalized by oligonucleotide fingerprinting so are not directly representative of relative EST expression. Three more genes were detected by RT-PCR in adult tissue. The low EST count of most GFPs suggests that other GFPs may be expressed below the level of detection of these EST libraries. In agreement with the heterozygosity of the genome sequence, most ESTs and RT-PCR products have multiple AA substitutions compared with the genomic sequences that are supported by multiple reads from different libraries.

Further evidence that these genes are functional comes from Ka/Ks ratios that show that all continue to be under clear purifying selection. Ka/Ks ratios for all amphioxus GFPs are less than one, by either pairwise or tree-based comparisons, and all but two comparisons are ≤0.5 (Additional file 1).

Twenty-one GFP-like sequences from the related *B. lanceolatum *are available in the NCBI patent database, of which 16 were full length. Putative alleles (>95% AA identity) were discarded, leaving 10 candidate distinct genes. These all mapped to clade b, but did not have distinct 1:1 orthologs in *B. floridae*, suggesting that this clade is evolving and expanding in both lineages.

We searched for homologs of these genes in the genomes of *Ciona intestinalis*, *C. savigyni *(ascidians), *Strongylocentrotus purpuratus *(purple sea urchin), and in the public nucleotide (NT), EST, and protein databases using profile HMMs and Blast. The closest homologs found were those previously described in copepod crustaceans, indicating that amphioxus is still the only deuterostome known to encode GFP-like proteins. The high similarity of amphioxus GFPs to each other and the spotty phylogenetic distribution of GFPs suggest that amphioxus acquired a single ancestral GFP by horizontal transfer, followed by extensive duplication and diversification.

### Spectral characteristics can be different among GFPs of amphioxus

**Figure 3:**
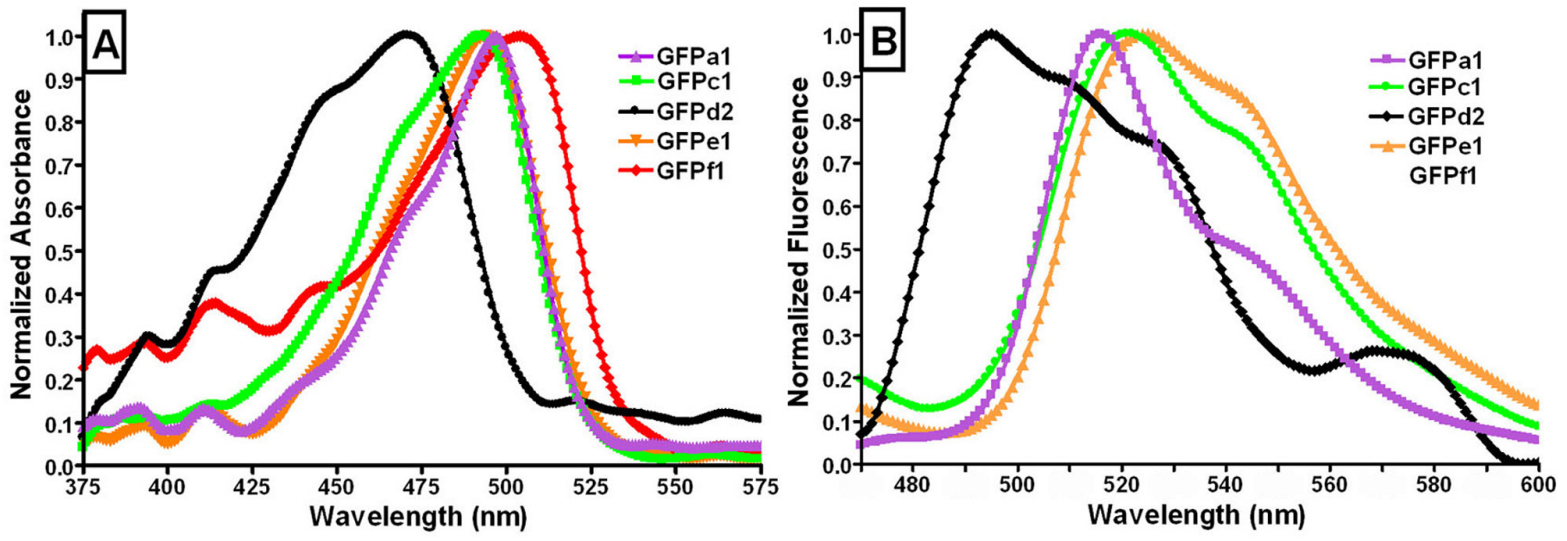
**Optical characteristics of amphioxus GFPs representatives of five clades**. A. Normalized absorbance spectra, B. Normalized fluorescent spectra. For both A and B, color scheme is coordinated with clades as in Figure 1.

We amplified, cloned, expressed, purified, and measured fluorescence and absorbance spectral characteristics of one representative of each clade, with the exception of clade b (GFPa1, GFPc1, GFPd2, GFPe1, and GFPf1). All proteins were well expressed, highly soluble, and easily purified. GFPa1, GFPe1, GFPd2, and GFPc1 showed monophasic absorbance and fluorescence spectra with more or less pronounced shoulders (Fig. 3), while GFPf1 was essentially non-fluorescent. The absorbance spectrum was different among GFPs showing a peak ranging from 470 to 504 nm (Table 2, Fig. 3A). Spectra of GFPa1 and GFPe1 were very similar with strongly overlapping profiles, while the spectrum was broader towards lower wavelengths for GFPc1, and shifted to lower wavelengths for GFPd2. Concentrated protein solutions of GFPc1, GFPd2, GFPe1, and GFPf1 were bright yellow in color while solutions of GFPa1 appeared greenish. Accordingly, the GFPs have very different extinction coefficients, ranging from 6,100 to 130,700 M^-1 ^cm^-1 ^at 500 nm (Table 2). This broad range encompasses the coefficient of 56,600 M^-1 ^cm^-1 ^found for the commercially available eGFP [29], which highlights the necessity to further investigate the photophysical characterization of the GFPs from amphioxus.

**Table 2:** Fluorescence and absorbance properties of amphioxus GFPs representative of five clades.

**GFP**	**Abs.** **Max** **(nm)**	**FWHM (nm)**	**Ex.** **Max (nm)**	**FWHM (nm)**	**Em. Max (nm)**	**FWHM (nm)**	**Extinction Coefficient (M^-1 ^cm^-1^) at 470 nm**	**Extinction Coefficient (M^-1 ^cm^-1^) at 500 nm**	**Relative Fluorescence Intensity**
GFPa1	497	45	500	38	516	39	48,100	120,900	100%
GFPe1	492	51	n.d.	n.d.	524	56	56,000	130,700	0.31%
GFPf1	504	60	n.d.	n.d.	n.f.	n.a.	13,400	25,500	n.a.
GFPd2	470	68	n.d.	n.d.	495	57	60,600	6,100	0.35%
GFPc1	493	55	n.d.	n.d.	521	56	63,900	98,800	0.33%

Excitation maximum was determined for emission set at 516 nm. Emission maximum was determined upon excitation at 500 nm for GFPa1 and at 465 nm for GFPe1, GFPf1, GFPd2, and GFPc1. Relative fluorescence intensity values are shown relative to the most fluorescent GFP, GFPa1. Abbrev.:Abs., absorbance, Ex., Excitation, Em., Emission, FWHM; full width half max, n.f., non-fluorescent; n.a., not applicable.

The fluorescence emission spectrum upon blue light excitation differed among GFPs, with peaks ranging from 495 to 529 nm (Table 2, Fig. 3B.). GFPf1 did not exhibit any detectable fluorescence. The GFPe1 and GFPc1 spectra were similar; GFPa1 was the sharpest, showing a pronounced shoulder at 540 nm while GFPd2 was much broader with developed shoulders at 510 and 525 nm showing an overall shift towards the blue (Fig. 3B).

The GFPa1 excitation spectrum for emission fixed at 516 nm peaked at 500 nm while spectra for GFPe1, GFPf1, GFPd2, and GFPc1 were below detection limits of the spectrophotometer.

In contrast to the somewhat subtle differences in spectral shape, the GFP-like proteins showed striking difference in fluorescent intensity (Table 2). GFPa1 exhibited the most intense fluorescence while the fluorescence intensity of the other proteins was about 200 times lower.

### Antioxidant activity associated with select amphioxus GFPs

**Figure 4:**
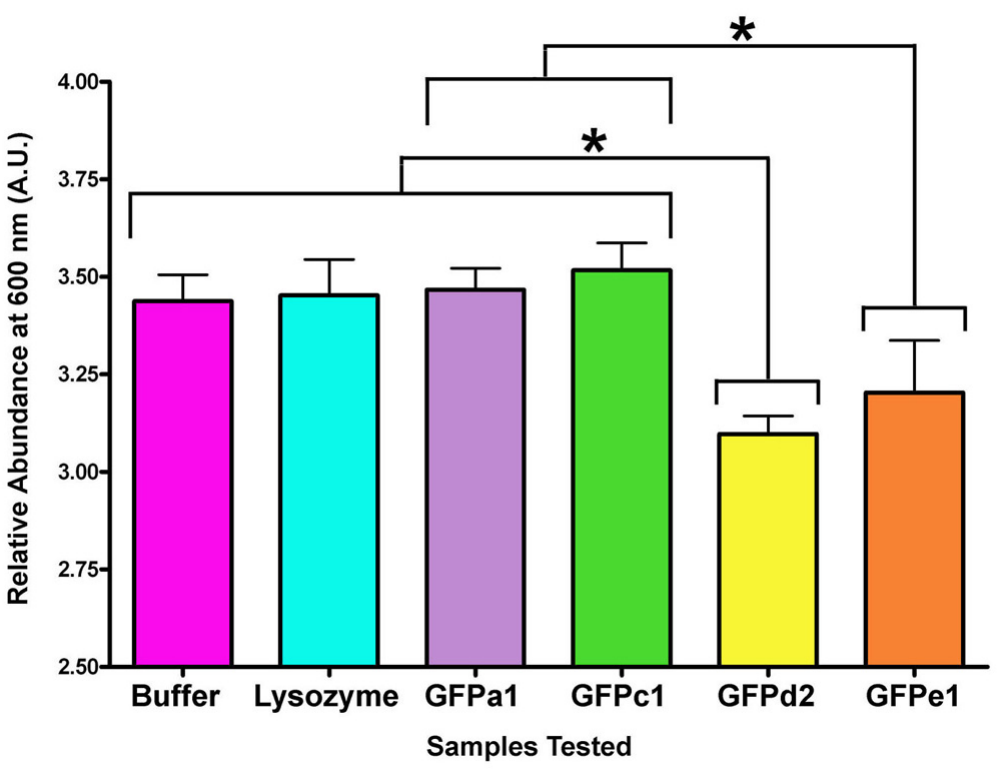
**Antioxidant capacity of amphioxus GFPs representative of four clades**. Statistically significant differences (*) supported by P < 0.05. Color scheme is coordinated with clades as in Figure 1.

GFPa1, GFPe1, GFPd2, and GFPc1 were evaluated for antioxidant capacity using the total antioxidant status assay (EMD Biosciences) whereby ABTS radical is formed from the reaction of ferrylmyoglobin radical and ABTS. The addition of antioxidants inhibits the formation of ABTS radical by quenching either the ferrylmyoglobin radical or the ABTS radical directly. GFPd2 and GFPe1 both showed antioxidant activity by substantially inhibiting formation of ABTS radical (Fig. 4). The GFPd2 activity was statistically significant (P < 0.05) relative to controls and inactive GFPs, while GFPe1 was borderline significant with P = 0.053, likely due to the small sample size (N = 3). In contrast, neither the highly fluorescent GFPa1 nor the weakly fluorescent GFPc1 showed any significant antioxidant activity (Fig. 4).

## Discussion

### Origin and evolution of amphioxus GFP

All *Branchiostoma floridae *GFPs are highly similar in sequence and genomic structure, and most are found in tandem duplications. Coupled with the apparent independent expansion of Clade b in *B. lanceolatum*, and the absence of GFP from any other deuterostome genomes or expressed sequences, this suggests that a single ancestral GFP was horizontally transferred into the amphioxus lineage, and then expanded by tandem duplication and transposition to form the current set of 16 genes. Accordingly, we showed here that all GFPs from *B. floridae *clustered together, suggesting a single horizontal acquisition from a common ancestor, which appears to be more closely related to copepod than cnidarian [25]. Ka/Ks ratios indicate that all of these genes are under purifying selection. There are substantial differences between alleles, between genomic and EST sequences, and between *B. floridae *and *B. lanceolatum *genes, suggesting that the GFP repertoire is continuing to diversify and evolve in amphioxus. The recent origin and high sequence similarity between GFPs with diverse properties suggests this as a model to dissect the sequence-function relationship within GFP.

### Distinct and dynamic developmental expression patterns of GFPs indicate functional diversity

We report here the occurrence of 6 clades of GFP-like proteins in amphioxus, encompassing 16 sequences. The strong EST expression and fluorescence of genes like GFPa1, indicates that light absorbance and/or fluorescence must serve an important function at some developmental or physiological stage. Unfertilized eggs show bright green autofluorescence that persists throughout the first cleavages of development after fertilization [30]. Even though the level of fluorescence appears variable through the next stages of development, it is clearly brighter again at the neurula stage [30], and spreads into bright green fluorescent patches throughout the larvae, though already appearing more concentrated around the buccal area, as for adults [25]. Such dynamic fluorescence display often reported throughout development in amphioxus is clearly supported here by the variable levels of expression for different GFPs (Fig. 2).

The whitish/transparent flesh color of adult amphioxus argues against a role for GFP in coloration (pigmentation) of mature amphioxus, correlating with the low expression seen in EST libraries. Amphioxus eggs have a yellow/cream color, which progressively fades to fully translucent from neurula onwards (Holland LZ, pers. comm.). This may be in part due to high expression of the GFPe clade in the earliest stages, though the later moderate expression of these genes in neurula and larva does not correlate with body color and may instead be involved in radical scavenging. Neurula and larva however are also brightly fluorescent, thus correlating with the expression of GFPa genes at these stages. The absence of other GFPs from these libraries may be due to highly selective expression within particular tissues, stages or physiological responses. This is indicated for example by the distinct patches of fluorescence in the larvae. Due to incompleteness and bias in EST libraries, this area warrants further exploration. Given, for instance, the selective expression of GFPa1 in the oral cirri where bright fluorescence is confined in adults [25], it will be valuable to explore expression of the various GFPs through *in situ *studies in order to address their specific function(s).

We propose that the six clades of GFP-like proteins may represent several functional classes of proteins with multiple variants in each class. Differential expression of members of each clade may then be coordinated to fulfil a given function with GFP-like expression varying temporally, spatially, and in concert with environmental changes.

### Distinct spectral characteristics among GFPs indicative of functional diversity

The spectral characteristics of each tested GFP show significant differences and suggest distinct functions for each GFP, or perhaps clade. The absorbance spectra have intriguing differences. In particular, GFPd2 lacks the typical GFP-500 nm peak retaining only a ~470 nm peak which is seen as a shoulder in GFPa1, GFPc1, and GFPe1 and has been previously described as a vibrational structure, bearing no electronic difference from the 500 nm-absorbing chromophore [31]. In agreement with Baumann et al. (2008), we find that GFPa1 exhibits remarkably bright fluorescence and is likely to be the protein responsible for fluorescence in the oral cirri. By contrast, GFPe1, GFPc1, and GFPd2 fluoresce weakly yet but are highly absorbent (high extinction coefficients) and may function in pigmentation during development, and/or in photoprotection. GFPf1 has a small extinction coefficient and no detectable fluorescence and so appears to lack a light-associated function. Whether the clades grouping GFPs based on protein sequence identity also correspond to groups of GFPs within distinct optical characteristics cannot be answered here, since only one representative GFP was analyzed per clade. However, the average of about 10% difference in sequence identity among GFPs from the same clade suggests that optical variability is possible within GFPs of the same clade [3,32]. Nevertheless, such diversity in optical characteristics from GFPs co-occurring in the same individual is probably the best indicator of multiple functions being explored within the family of amphioxus GFPs.

### Some amphioxus GFPs exhibit a highly divergent chromophore site

Delagrave and colleagues have shown that the glycine in the third position of the chromophore triplet is essential for chromophore formation [33]. No chromophore bearing other than the XXG template exists in natural or engineered GFP-like proteins [34]. Like their closest relatives, copepods, nearly all the amphioxus GFP-like proteins have a GYG triplet. The only exception, and the first non-XXG GFPs yet seen are in the two alleles of both GFPb1 and GFPb2, which contain a GYA triplet. All Clade b genes in *B. lanceolatum *retain the GYG triplet, suggesting that the GYA is a recent evolutionary innovation.

### Expansion of GFP gene family in amphioxus may bolster cellular defense via antioxidant activity

Gene family expansions as seen for GFP are relatively rare in amphioxus, but are enriched for specific functions, including cellular defence [35]. We speculate that the antioxidant function of some GFPs may fall into this category and function by scavenging deleterious oxy-radicals. Bou-Abdallah and colleagues have shown *in vitro *that wild type GFP from *Aequorea victoria *quenches superoxide radicals (O_2_^•-^) and exhibits SOD-like activity by competing with cytochrome *c *for reaction with O_2_^•- ^[22]. We have shown here that at least two amphioxus proteins, GFPe1 and GFPd2, have antioxidant activity. GFPe1 shows optical characteristics different than GFPd2, being similar to GFPa1 (fluorescent) and GFPc1 (essentially non-fluorescent), which lack antioxidant activity. Thus the antioxidant and optical properties do not correlate.

## Conclusion

We have described the largest known family of GFP proteins and the only known deuterostome GFPs. Despite their high sequence similarity, they have significant spectroscopic and functional differences. Amphioxus contains a GFP that is highly fluorescent, GFPs that are weakly or essentially non-fluorescent, bearing either high or low extinction coefficients, and GFPs that are weakly fluorescent yet bear antioxidant activity, suggesting the existence of at least four functional classes of amphioxus GFPs (Table 3). All GFPs are also under selective pressure, and show a wide range of distinct expression patterns, supporting the suggestion that all have distinct functions in the animal.

**Table 3:** Summary of optical and biochemical properties of a representative GFP from each amphioxus GFPs clade.

	**Amphioxus GFP clade**					
	
	a	b	c	d	e	F
Representative GFP	GFPa1	n.d.	GFPc1	GFPd2	GFPe1	GFPf1
						
**Characteristic**						
Fluorescence	Strong	n.d.	Weak	Weak	Weak	None
Absorbance	High	n.d.	High	Moderate-high	High	Low
Antioxidant	No	n.d.	No	Yes	Yes	n.d.

Abbrev.: n.d.: not determined

While the precise roles of these proteins remains unresolved, this work establishes clearly that amphioxus GFPs have multiple light-associated and light-independent functions. The divergence of GFPs from *B. lanceolatum *and the conserved sequences and genomic organization of amphioxus GFPs suggest that future cloning and characterization of GFPs from multiple amphioxus species, and engineering of chimeras will be a feasible and productive method to better understand the evolution and sequence-function relationship for this first family of deuterostome GFPs.

## Methods

### Sequence Analysis

GFP-like sequences were predicted from Assembly 1 of the amphioxus genome [36] and gene predictions, using Blast. Alleles were distinguished by inspection of intronic sequence conservation, mapping to Assembly 2, and identity of neighboring genes. Ka/Ks ratios were calculated using DnaSp [37] and an online server [38]. Searches for GFPs in other sequence sources used Blast, Psi-Blast, HMMer and Gene Detective, a hardware implementation of the Genewise algorithm (Active Motif, Carlsbad, CA). ESTs were downloaded from NCBI on Nov 12, 2008, and Blasted against GFP nucleotide sequences. Library information was extracted from EST FastA headers.

### RNA Extraction and Protein Cloning

*Branchiostoma floridae *(amphioxus), collected from Tampa Bay, Florida were frozen at -80°C, ground to a paste using a mortar and pestle and RNA extracted using TriReagent (Sigma) and the RNeasy kit (Invitrogen, Carlsbad, California) according to the manufacturer's protocol. cDNA was made with the Retroscript kit (Ambion, Austin, Texas). Gene-specific primers for GFPa1, GFPc1, GFPd2, and GFPe1 were designed based on sequences from the genome database (JGI) and used to amplify GFP-like genes from amphioxus cDNA by polymerase chain reaction (PCR). Gene-specific primers for GFPf1 were designed based on sequences from the genome database (JGI) and used to amplify GFPf1 from amphioxus EST clone CAXG1077. PCR products were cloned into either the pET24b(+) *E. coli *expression vector (Novogen, New Canaan, Connecticut) at NdeI and HindIII restrictions sites or the pHIS8 *E. coli *expression vector at NcoI and HindIII restrictions sites [39].

### Protein Expression and Purification

Transformed *E. coli *BL21(DE3) cells were incubated with shaking at 37°C in Luria broth [40] containing 50 μg/ml kanamycin until OD_600 nm _= 0.8. Protein expression was induced with 0.5 mM isopropyl 1-thio-β-galactopyranoside (IPTG) and the cultures were incubated with shaking at 37°C for 4 hours. Cells were harvested by centrifugation at 9,000 g and cell pellets resuspended in lysis buffer [400 mM NaCl, 50 mM Tris-HCl (pH 8.0), 10% glycerol, 10 mM BME] supplemented with 0.5 mg/ml lysozyme. Following sonication and centrifugation at 100,000 g, supernatant was passed over a Ni^2+^-NTA column (Qiagen, Valencia, CA) equilibrated in lysis buffer, washed with 10 bed volumes of wash buffer [400 mM NaCl, 50 mM Tris-HCl (pH 8.0), 20 mM imidazole, 10 mM BME] and the His-tagged protein eluted with 10 bed volumes of elution buffer [400 mM NaCl, 50 mM Tris-HCl (pH 8.0), 250 mM imidazole, 10 mM BME]. Subsequently, 30 units of thrombin protease were added and the eluted protein was dialyzed overnight against 400 mM NaCl, 50 mM Tris pH8, 10 mM BME. The retentate was reloaded onto a Ni^2+^-NTA column to remove the protein bearing uncleaved His-tags and then passed over a benzamidine sepharose column to remove the thrombin protease. The resulting flowthrough was concentrated and loaded onto a Superdex S200 gel filtration column (Amersham, Piscataway, New Jersey) equilibrated in gel filtration buffer [400 mM NaCl 50 mM TRIS-HCl (pH 8.0), 1 mM DTT] to isolate homogeneous dimeric GFPa1, GFPc1, GFPd2, GFPe1, and GFPf1. Peak fractions were collected, concentrated, and stored at -80°C.

### Spectral Characterization

Fluorescence spectra were recorded with a SE200 low-light digital spectrograph (Catalina Scientific Instruments, Tucson, AZ) upon excitation with a 465 nm LED (Ocean Optics, Dunedin, FL) that showed a narrow spectrum as defined by the Full Width at Half the Maximum intensity (FWHM = 31 nm). To measure fluorescence spectra GFP-like proteins were diluted to 1 μM and excitation was set at 800 msec and zero electronic gain. All dilutions were done with gel filtration buffer. Normalized fluorescence spectra were measured with proteins diluted to 1 μM (GFPa1) or 10 μM (GFPc1, GFPd2, GFPe1, and GFPf1). Excitation was set at 800 msec and zero electronic gain (GFPa1) or 5 sec with 150 electronic gain (GFPe1, GFPc1, GFPd2, and GFPf1). Absorbance spectra were recorded with the same apparatus. Normalized absorbance spectra were recorded from proteins diluted to 10 μM. A Spectra Max M2 (Molecular Devices, Sunnyvale, CA) spectrophotometer was used to measure excitation spectra at 10 μM protein concentration. Fluorescence emission was set at 515 nm while excitation was scanned from 350–505 nm.

### Extinction Coefficient

Protein concentrations were calculated using the extinction coefficient of the chromophore after denaturation in 0.1 N NaOH (44, 000 M^-1 ^cm^-1 ^at 446 nm) [41,42].

Absorbance of GFPa1, GFPc1, GFPd2, GFPe1, and GFPf1 was measured using a Spectra Max M2 (Molecular Devices, Sunnyvale, CA) spectrophotometer and extinction coefficients calculated according to the Beer Lambert law.

### bfloGFP Antioxidant Capacity

The total antioxidant status assay (EMD Biosciences, Darmstadt, Germany) was used to evaluate antioxidant activity. This assay is based upon the inhibition of the ferrylmyoglobin^•^-catalyzed oxidation of colorless ABTS (2,2'-azino-bis(3-ethylbenzthiazoline-6-sulphonic acid) into ABTS^• ^which is green in color and absorbs at 600 nm. Addition of antioxidant quenches the ferrylmyoglobin^•^, inhibits ABTS^• ^formation, and decreases absorbance at 600 nm. GFPs do not absorb or fluoresce in the 600 nm range, and so they do not optically interfere in this assay. Total antioxidant status assay reactions were carried out according to manufacturer's protocol. Briefly, 20 μL of either sample buffer, a protein sample, or the reaction standard, 6-Hydroxy-2,5,7,8-tetramethylchroman-2-carboxylic acid, was added to 1 ml of phosphate buffered saline containing metmyoglobin and ABTS and vortexed. 200 μL of H_2_O_2 _substrate was added, vortexed, and the absorbance at 600 nm was followed for 3 minutes. All proteins were diluted to 200 μM prior to addition. Addition of GFP buffer alone (50 mM Tris pH 8.0, 400 mM NaCl, 1 mM DTT) served as a control reaction while addition of lysozyme (Sigma, St. Louis, Missouri) served as a negative control. Absorbance values were expressed relative to the reaction standard. The assay measurements were completed three independent times (N = 3). At the end of the assay, OD values from each sample measurement were expressed relative to the mean OD obtained from the standard. Relative OD values were then log(x+1) transformed to respect homocedasticity of the statistical analysis (Zar, 1996). Differences among samples were tested for statistical significance with α of 0.05 for two-tailed comparisons. Analysis of Variance (ANOVA) and post-hoc multiple comparison of means (Fisher's PLSD) were used to test differences, using Statview 5.0 (SAS Institute, Inc.).

## Authors' contributions

EKB carried out the molecular, biochemical and photonic studies, initiated the bioinformatics analysis and drafted the manuscript. GM carried out the genomic and bioinformatic in-depth analyses and contributed to development of the manuscript. DDD conceived the study, and participated in its design and coordination, and contributed to development of the manuscript. All authors read and approved the final version of the manuscript.

## Supplementary Material

Additional file 1 **Zip file containing predicted cDNA and protein sequences for all GFP sequences including alleles, and Ka/Ks substitution rates calculated using pairwise and tree-based methods.** Click here for file
