# Supplementary figures and images for: Amphioxus encodes the largest known family of green fluorescent proteins, which have diversified into distinct functional classes
Source: BMC Evol Biol. 2009 Apr 21;9:77. doi: 10.1186/1471-2148-9-77 (PMC2679011; doi:10.1186/1471-2148-9-77)

Ka/Ks annotated evolutionary tree

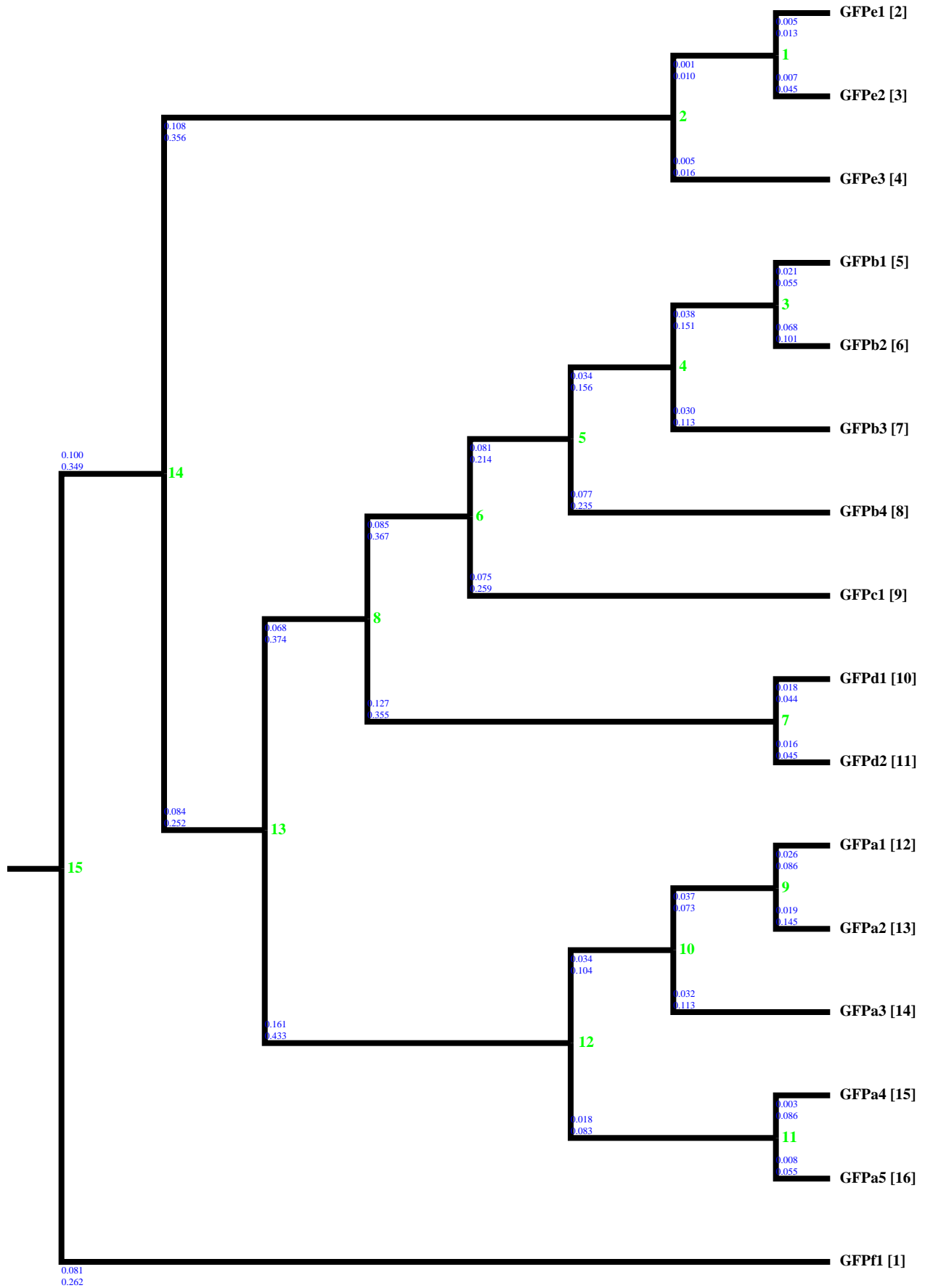

Supplement: Additional file 1 — Zip file containing predicted cDNA and protein sequences for all GFP sequences including alleles, and Ka/Ks substitution rates calculated using pairwise and tree-based methods. [file 1471-2148-9-77-S1.zip › Bomati etal_SOM package/kaks_tree.pdf]
